# Multicenter Clinical Evaluation of a Novel Multiplex Real-Time PCR (qPCR) Assay for Detection of Fluoroquinolone Resistance in Mycoplasma genitalium

**DOI:** 10.1128/JCM.00886-19

**Published:** 2019-10-23

**Authors:** Miguel Fernández-Huerta, Kaveesha Bodiyabadu, Juliana Esperalba, Catriona S. Bradshaw, Judit Serra-Pladevall, Suzanne M. Garland, Candela Fernández-Naval, Jorgen S. Jensen, Tomàs Pumarola, Samantha Ebeyan, Marie Lundgren, Lit Yeen Tan, Mateu Espasa, Gerald L. Murray

**Affiliations:** a Microbiology Department, Vall d’Hebron University Hospital, Universitat Autònoma de Barcelona, Barcelona, Spain; b SpeeDx Pty Ltd, Sydney, Australia; c Centre for Women’s Infectious Diseases, The Royal Women’s Hospital, Parkville, Victoria, Australia; d Murdoch Children’s Research Institute, Parkville, Victoria, Australia; e Central Clinical School, Monash University, Melbourne, Australia; f Melbourne Sexual Health Centre, Alfred Health, Melbourne, Australia; g Department of Obstetrics and Gynaecology, University of Melbourne, Parkville, Victoria, Australia; h Statens Serum Institut, Copenhagen, Denmark; i Microbiology Department, Corporació Sanitària Parc Taulí, Universitat Autònoma de Barcelona, Barcelona, Spain; Marquette University

**Keywords:** *Mycoplasma genitalium*, fluoroquinolone resistance, multiplex qPCR assay, multicenter evaluation, antibiotic resistance

## Abstract

Mycoplasma genitalium causes a common sexually transmitted infection with a marked propensity to develop antimicrobial resistance. As few treatment options exist, this poses significant challenges to clinicians. Recent diagnostic advances have resulted in tests that report the simultaneous detection of M. genitalium and any resistance to macrolides, the first-line treatment. This allows for therapy to be tailored to the individual, thereby optimizing treatment outcomes. However, resistance to fluoroquinolones, the second-line treatment, is increasing in M. genitalium. In this study, we describe a new assay, MG+parC (beta), which simultaneously reports the detection of M. genitalium and five *parC* mutations that have been associated with resistance to fluoroquinolones. These mutations affect the amino acid sequence of ParC at residues S83R (A247C), S83I (G248T), D87N (G259A), D87Y (G259T), and D87H (G259C). The study tested the MG+parC (beta) assay with 202 M. genitalium-positive clinical samples from Australia (*n* = 141) and Spain (*n* = 61). Compared to Sanger sequencing, the assay performed with a kappa value of 0.985 (95% confidence interval [CI], 0.955 to 1.000), with a mutation detection sensitivity of 97.6% (95% CI, 87.4 to 99.9), and specificity of 100.0% (95% CI, 97.7 to 100.0). Fluoroquinolone resistance-associated mutations in *parC* targeted by the assay were more prevalent among the Australian cohort (23.4% [95% CI,16.3 to 31.8]) compared to the Spanish population (8.8% [95% CI, 2.9% to 19.3%]) (*P = *0.019). The MG+parC (beta) kit is a simple and reliable method for simultaneous detection of M. genitalium and fluoroquinolone resistance-associated mutations in clinical settings. This novel diagnostic tool may extend the utility of the second line of antimicrobial therapies in M. genitalium infection.

## Abstract (precis)

Mycoplasma genitalium causes a common sexually transmitted infection with a marked propensity to develop antimicrobial resistance. As few treatment options exist, this poses significant challenges to clinicians. Recent diagnostic advances have resulted in tests that report the simultaneous detection of M. genitalium and any resistance to macrolides, the first-line treatment. This allows for therapy to be tailored to the individual, thereby optimizing treatment outcomes.

## INTRODUCTION

Mycoplasma genitalium causes a sexually transmitted infection (STI) that causes urethritis in men and is associated with a number of genitourinary complications in women, including pelvic inflammatory disease (PID) and preterm birth (1). M. genitalium has a highly reduced genome, which is responsible for a limited metabolic and structural complexity, making it susceptible to a very restricted range of antibiotics (2). Current international treatment guidelines recommend first-line treatment with the macrolide azithromycin (extended regimen) and second-line treatment with the fluoroquinolone moxifloxacin (3). The fluoroquinolone sitafloxacin is used in Japan (3, 4) and has been utilized by special import in Australia (5). However, resistance to both these classes of antimicrobials has rapidly emerged worldwide (6, 7).

While macrolide resistance is strongly associated with single-nucleotide polymorphisms (SNPs) at positions 2071 or 2072 of the 23S rRNA gene (8), fluoroquinolone resistance in M. genitalium is less well established. Mutations in the quinolone resistance-determining region (QRDR) of the *parC* gene (encoding subunit A of topoisomerase IV) have been associated with *in vitro* and *in vivo* resistance to “fourth-generation” fluoroquinolones, as moxifloxacin. In particular, several specific missense SNPs affecting serine 83 (S83) and aspartic acid 87 (D87), which play a key role in mediating quinolone-enzyme interactions (9), have been associated with moxifloxacin failure in M. genitalium in clinical studies (6, 10). These include the amino acid changes S83R, S83I, D87Y, D87H, and D87N, which have been identified in fluoroquinolone treatment failures (6, 10–12) or determined to have elevated fluoroquinolone MICs *in vitro* (13).

Since there are very few therapeutic alternatives available for M. genitalium infection, any strategy that prolongs the utility of existing treatments is extremely important. Recently, a resistance-guided sequential therapy approach utilizing a novel technology that reports both detections of the organism and macrolide resistance mutations (ResistancePlus MG; SpeeDx Pty Ltd, Australia) was prospectively evaluated (5, 14). This strategy, which allows the physician to prescribe the appropriate antibiotic in each case, resulted in a remarkable cure rate of more than 92% in a population with high levels of macrolide and fluoroquinolone resistance (5, 6). Furthermore, the selection of antibiotic resistance in macrolide susceptible infections treated with sequential therapy was minimized to less than 3% in the study (5). In this scenario, the success of antimicrobial resistance-guided treatment could be extended through the development of assays that also report the detection of fluoroquinolone resistance-associated mutations in M. genitalium.

In response to this challenge, a novel multiplex real-time PCR (qPCR) assay has been developed to detect the fluoroquinolone resistance-associated mutations in M. genitalium infections, which have been consistently associated with treatment failure following moxifloxacin, namely, G248T (S83I), A247C (S83R) G259T (D87Y), G259A (D87N), and G259C (D87H). The primary objective of this study was to evaluate this assay (MG+parC beta; SpeeDx Pty Ltd, Australia) using two geographically and clinically distinct populations. The performance of the assay was compared to the gold standard, Sanger sequencing of the *parC* gene. The study also reported the prevalence of specific mutations linked to fluoroquinolone resistance and treatment failure in these two distinct settings.

## MATERIALS AND METHODS

### Sample selection.

Samples from Australia were collected at the Melbourne Sexual Health Centre, as part of a resistance-guided treatment study (5), from June 2016 to May 2017. From 167 patients with macrolide-resistant M. genitalium infection who were undergoing sitafloxacin treatment, the analysis was performed on 126 baseline samples and 15 test-of-cure (TOC) samples from 124 individuals (a total of 141 specimens). Samples included 85 first-void urine samples, 30 rectal swabs, 18 vaginal swabs, 7 endocervical swabs, and 1 urethral swab. Demographic characteristics for the parent study group were published elsewhere (5). Comprehensive analysis of the association between specific ParC and GyrA mutations and sitafloxacin cure is the subject of another study and is not presented here.

Samples from Spain were processed at the Vall d’Hebron University Hospital in Barcelona as part of another study (15). Samples were collected from asymptomatic individuals attending an STI screening service (DraSexp) from October 2017 to January 2018. A total of 61 M. genitalium-positive baseline specimens from 57 participants were included in the evaluation. Samples consisted of 16 first-void urine samples, 38 self-collected rectal swabs, and 7 self-collected vaginal swabs.

Samples were stored at –80°C prior to use, and DNA was stored at –20°C to –30°C.

Ethical approvals for the study were obtained from the Vall d’Hebron University Hospital Ethics Committee in Spain (approval number 209/17) and the Royal Women´s Hospital Research and Ethics Committees and the Alfred Hospital Ethics Committee in Australia (approval number 232/16).

### Laboratory procedures.

Samples were initially tested using the ResistancePlus MG test according to the manufacturer’s instructions. Briefly, 200 μl of sample was extracted using the MagNA Pure 96 instrument (Roche Diagnostics, USA) and eluted in a final volume of 100 μl with the DNA and Viral NA small-volume extraction kit using the Pathogen Universal 200 protocol (Roche Diagnostics, USA). Then, 5 μl of DNA was amplified in a 20-μl reaction volume. Positive samples were then included in the fluoroquinolone resistance assay evaluation study.

M. genitalium-positive samples were tested with the MG+parC (beta) assay as described by the manufacturer. A 5-μl aliquot of DNA extract was added to each of two wells, each containing a 15-μl reaction mix (10 μl Plex Mastermix, 1.0 μl of MG+parC mix 1 or 2, and 4.0 μl nuclease-free water), for a final reaction volume of 20 μl, tested in 96 well plates. The PCR was performed on the LightCycler 480 Instrument II (Roche Diagnostic, USA). Cycling consisted of 95°C for 2 min (ramp speed, 4.4°C/s), followed by 10 cycles of 95°C for 5 s and 61°C for 30 s (−0.5°C per cycle; ramp speed, 2.2°/s), 40 cycles of 95°C for 5 s (ramp speed, 4.4°C/s), and 52°C for 40 s (ramp speed, 2.2°C/s), followed by cooling (4°C, 30-s hold; ramp speed, 2.2°C/s). Data acquisition was set for channels A (465 to 510 nm) and B (533 to 610 nm). Analysis of the results was performed using the supplied software.

Sanger sequencing was performed as the comparator assay. PCR amplicons were generated with primers MG-*parC*124-F (5′-AAACCAGTACAAAGACGGATCT-3′) and MG-*parC*478-R (5′-GAGGTTAGGCAGTAAGGTTGG-3′). For samples that primarily failed to amplify, internal primers MG-*parC*-A (5′-TGGGCTTAAAACCCACCACT-3′) and MG-*parC*-B (5′-CGGGTTTCTGTGTAACGCAT-3′) were used to perform a nested PCR (11). An M. genitalium G37 published genome was used as a reference (16).

### Description of the MG+parC beta assay.

The MG+parC (beta) test is a new two-well qPCR assay that utilizes PlexZyme and PlexPrime technology to enable a high degree of multiplexing (17, 18). The assay detects M. genitalium through the *mgpB* gene (MG191) in readout 1 of wells 1 and 2 (6-carboxyfluorescein [6-FAM] dye [excitation wavelength {λ_ex_}, 495 nm; emission wavelength {λ_em_}, 516 nm]). Simultaneously, via readout 2, the assay detects the *parC* mutation G248T (Texas Red [λ_ex_, 553 nm; λ_em_, 610 nm]) in well 1 and the presence of A247C, G259A, G259T, or G259C mutations (Texas Red [λ_ex_, 553 nm; λ_em_, 610 nm]) in well 2.

### Statistical analyses.

The clinical sensitivity and specificity for detection of fluoroquinolone-resistance associated ParC mutations in M. genitalium for the MG+parC (beta) assay were calculated by comparison with Sanger sequencing of the *parC* gene. Additionally, the kappa statistic (κ) was used to evaluate the agreement between the MG+parC (beta) test and Sanger sequencing. Statistical analyses were performed with Stata (StataCorp, USA). Distributions of categorical variables were compared by Chi-square (χ^2^) or Fisher’s exact tests. The 95% confidence intervals (CI) were calculated by exact methods and differences, with a *P* value of *<*0.05 considered statistically significant.

## RESULTS

### Evaluation of the MG+parC (beta) assay on clinical specimens.

A total of 202 specimens were evaluated using the MG+parC (beta) assay in comparison to Sanger sequencing. Results are displayed in Table 1, separated by cohorts. In a combined analysis, the level of concordance of the MG+parC (beta) assay with the reference method was 99.5% (95% CI, 97.3 to 100.0), with a kappa value of 0.985 (95% CI, 0.955 to 1.000). Additionally, overall sensitivity and specificity were 97.6% (95% CI, 87.4 to 99.9) and 100.0% (95% CI, 97.7 to 100.0), respectively. Notably, the only discordant sample [G259T (D87Y), reported as nonmutant by the MG+parC (beta) commercial assay], was found among the specimens from Australia and was unavailable for discrepant analysis.

**TABLE 1:** Evaluation of mutation detection by the MG+parC (beta) assay compared to Sanger sequencing of the *parC* gene^*e*^

Sample origin (*n*)	MG+parC (beta) assay	Sanger sequencing results (no.)		Sensitivity (% [95% CI])	Specificity (% [95% CI])
		Nonmutant	Mutant^*a*^		
Spain (61)	Nonmutant	56	0	100.0 (47.8–100.0)	100.0 (93.6–100.0)
	Mutant	0	5^*b*^		
Australia (141)	Nonmutant	104	1^*c*^	97.3 (85.8–99.9)	100.0 (96.5–100.0)
	Mutant	0	36^*d*^		

a Mutant category includes mutants targeted in the MG+parC (beta) assay with SNPs A247C (S83R), G248T (S83I), G259T (D87Y), G259C (D87H), and G259A (D87N).

b The sample set included 2 G248T (S83I) and 3 G259T (D87Y) mutants.

c Mutant G259T (D87Y) was reported as nonmutant by the commercial assay. The sample was unavailable for discrepant analysis.

d The sample set included 32 G248T (S83I), 2 G259A (D87N), and 2 G259T (D87Y).

e CI, confidence interval.

### Prevalence of fluoroquinolone resistance-associated mutations in *parC*.

The characteristics of the 57 individuals studied in the Spanish cohort are described in Table 2. Overall, the prevalence of fluoroquinolone resistance-associated mutations targeted in the MG+parC (beta) assay was 8.8% (95% CI, 2.9% to 19.3%). Mutants consisted of 2 G248T (S83I) and 3 G259T (D87Y). All infections harboring these potential resistance mutations occurred in men who have sex with men (MSM). Additionally, 2 samples had SNPs in position G248A (S83N), which are not targets of the current assay but will be present in a subsequent version.

**TABLE 2:** Prevalence of *parC* fluoroquinolone resistance-associated mutations in M. genitalium infections in Spain^*e*^

Patient characteristics	M. genitalium			
	*parC* mutant^*a*^		Nonmutant	
	*N*; %	95% CI	*N*; %	95% CI
Spanish cohort (*n* = 57)	5^*b*^; 8.8	2.9–19.3	52^*c*^; 91.2	80.7–97.1
Sexual preference^*f*^				
WSM	0; 0.0	0.0–52.2	10; 19.2	9.6–32.5
MSW	0; 0.0	0.0–52.2	9; 17.3	8.2–30.3
MSM	5; 100.0	47.8–100.0	33; 63.5	49.0–76.4
HIV status				
Positive	1; 20.0	0.5–71.6	9; 17.3	8.2–30.3
Negative	4; 80.0	28.4–99.5	43; 82.7	69.7–91.8
Macrolide resistance status				
Resistant	4; 80.0	28.4–99.5	26; 50.0	35.8–64.2
Susceptible	1; 20.0	0.5–71.6	26; 50.0	35.8–64.2
Location				
Genital	1; 20.0	0.5–71.6	22^*d*^; 42.3	28.7–56.8
Rectum	4; 80.0	28.4–99.5	34; 65.4	50.9–78.0

a Mutant category includes the mutants targeted in the MG+parC (beta) assay—A247C (S83R), G248T (S83I), G259T (D87Y), G259C (D87H), and G259A (D87N)—confirmed with Sanger sequencing.

b Mutants included 2 G248T (S83I) and 3 G259T (D87Y).

c Nonmutant infections included 2 with missense SNPs in position G248A (S83N).

d Four patients (3 MSM and one woman) had both genital and rectal M. genitalium infections.

e CI, confidence interval.

f MSW, men who have sex with women; MSM, men who have sex with men; WSM, women who have sex with men.

The prevalence of ParC fluoroquinolone resistance-associated mutants targeted in the commercial assay among the 124 participants (baseline samples) screened in the Australian cohort was 23.4% (95% CI, 16.3 to 31.8), including 25 G248T (S83I), 2 G259T (D87Y), and 2G259A (D87N). Additionally, there were 3 samples with mutations present that are not targeted by the assay, A247T (S83C), T249G (S83R), and G248A (S83N).

## DISCUSSION

This study is an evaluation of the MG+parC (beta) diagnostic assay, which detects five M. genitalium mutations associated with fluoroquinolone resistance (6, 10, 11, 13). The performance was analyzed in two distinct settings, Australia and Spain, involving both symptomatic and asymptomatic men and women and specimens of M. genitalium coming from different anatomical sites (first-void urine samples, rectal swabs, vaginal swabs, endocervical swabs, and urethral swabs). For all patients with multisite infections, ParC results were concordant within the patient. Overall, the MG+parC (beta) kit performed very well (kappa value of 0.985) for detection of fluoroquinolone resistance-associated mutations compared with Sanger sequencing of the *parC* gene. The high level of performance suggests that there was no issue with sample degradation during storage. The single discordant sample was unavailable for discrepant analysis. No false-positive results were reported in the evaluation.

Unlike macrolide resistance in M. genitalium, the presence of missense SNPs in *parC* do not always translate into treatment failures with fluoroquinolones, and the data associating a number of these mutations with clinical outcomes are quite limited (6, 10, 13). However, there is a clear need to develop diagnostic tests that will detect fluoroquinolone resistance and optimize the first-line selection of antimicrobials (14). So, the novel MG+parC (beta) assay includes detection of three fluoroquinolone resistance mutations that have been consistently associated with treatment failure following moxifloxacin, the most widely available fluoroquinolone in use for M. genitalium, namely, G248T (S83I), A247C (S83R), and G259T (D87Y) (6, 10, 13), and also included the G259A (D87N) mutation, which has been associated with elevated moxifloxacin MICs in 3 strains (J. S. Jensen, personal communication) and G259C (D87H), for which there is some evidence for moxifloxacin resistance (10, 12), but this requires further investigation. Importantly, this current version of the test does not target another mutation in S83, G248A (S83N), which is being included in a newer version of the assay in a separate channel (SpeeDx Pty Ltd, personal communication), since this mutation is unlikely to contribute to moxifloxacin treatment failure as estimated from *in vitro* MICs from 4 isolates (19; J. S. Jensen, personal communication). However, it could help to monitor the development of additional mutations, and this addition may prove clinically relevant.

The present study included samples from two geographically and clinically distinct populations. The prevalence of fluoroquinolone resistance-associated mutations covered by the MG+parC (beta) assay was 23.4% in Australia and 8.8% in Spain (*P = *0.019). Including mutations not detected by the assay (S83N, the alternative T249G encoding S83R, and S83C—of unknown impact on antibiotic resistance) increased prevalence to 25.8% and 12.3%, respectively (*P = *0.040). Previously reported levels of ParC mutation were 13.6% at the Australian site for samples collected in 2012 and 2013 (6) and 8.3% at the Spanish site for samples collected in 2013 and 2014 (7). The study provides further evidence that fluoroquinolone resistance is already a major issue in the Asia-Pacific region, where the use of these agents is more common, while it is emerging in Europe, particularly among MSM (6, 7, 12). It should be noted that the Australian samples are from M. genitalium-infected patients with existing macrolide resistance mutations, and fluoroquinolone resistance mutations are more commonly detected in conjunction with macrolide resistance and are considered multidrug-resistant strains (6, 7).

The described qPCR assay is more efficient and potentially less expensive than performing Sanger sequencing, although, unlike Sanger sequencing, it has a limitation in that it detects only a defined set of mutations. The novel MG+parC (beta) assay does not require specialized instrumentation and can be easily implemented in routine diagnosis. The test offers the first commercial kit to date that is capable of simultaneously detecting M. genitalium and fluoroquinolone resistance-associated mutations in *parC*. Although the current version of this test detects the relevant mutations mentioned above, only G248T (S83I) in well 1, the mutation most consistently linked with fluoroquinolone resistance, is individually reported by the assay. The other SNPs, A247C (S83R), G259A (D87N), G259T (D87Y), and G259C (D87H), are reported in the same channel as resistant genotypes. Importantly, future assay development may have a revised format and include additional mutations (SpeeDx Pty Ltd, personal communication) according to ongoing and future resistance surveillance studies from our research group and others.

With the incipient introduction of assays detecting both M. genitalium and macrolide resistance mutations, the MG+parC (beta) test or future versions of this assay may also be used to further refine the diagnostic algorithms based on the resistance-guided therapy approach and, ultimately, to optimize antimicrobial stewardship (5, 20). While the ParC mutations included in this assay have been associated with moxifloxacin treatment failure and/or reduced susceptibility to moxifloxacin and/or sitafloxacin in *in vitro* studies, more data are needed to fully establish the contribution of specific ParC mutations to treatment outcomes with these agents. Furthermore, additional research is needed to find therapeutic alternatives against multidrug resistant infections (e.g., novel antimicrobials or combinations of existing treatments).

In conclusion, the MG+parC (beta) kit is a rapid, simple, and accurate assay for simultaneous detection of M. genitalium and fluoroquinolone resistance-associated mutations in clinical settings. The application of this novel diagnostic tool is likely to improve patient management in clinical practice in the future.
